# Transcription Factor *Pso9TF* Assists Xinjiang Wild Myrobalan Plum (*Prunus sogdiana*) *PsoRPM3* Disease Resistance Protein to Resist *Meloidogyne incognita*

**DOI:** 10.3390/plants10081561

**Published:** 2021-07-29

**Authors:** Haifeng Zhu, Kun Xiao, Wenjiang Pu, Zhenhua Liu, Yan Wang, Chaoyuan Gao, Sifang Luo, Yue Xu, Pingyin Guan, Jianfang Hu

**Affiliations:** Laboratory of Fruit Physiology and Molecular Biology, China Agricultural University, Beijing 100193, China; zhf51@outlook.com (H.Z.); xiaokun201@126.com (K.X.); puwj07@outlook.com (W.P.); liuzhenhua93@163.com (Z.L.); tracywang0104@163.com (Y.W.); CRISTIANOGAO7@163.com (C.G.); Sifang.Luo@outlook.com (S.L.); xuyueau@163.com (Y.X.); pyguan@cau.edu.cn (P.G.)

**Keywords:** Xinjiang wild myrobalan plum (*Prunus sogdiana*), *Meloidogyne incognita*, *PsoRPM3*, *Pso9TF*, hypersensitive response (HR), tobacco

## Abstract

The root-knot nematode (*Meloidogyne incognita*) causes huge economic losses in the agricultural industry throughout the world. Control methods against these polyphagous plant endoparasites are sparse, the preferred one being the deployment of plant cultivars or rootstocks bearing resistance genes against *Meloidogyne* species. Our previous study has cloned one resistance gene, *PsoRPM3*, from Xinjiang wild myrobalan plum (*Prunus sogdiana*). However, the function of *PsoRPM3* remains elusive. In the present study, we have investigated the regulatory mechanism of *PsoRPM3* in plant defense responses to *M. incognita*. Our results indicate that fewer giant cells were detected in the roots of the *PsoRPM3* transgenic tobacco than wild tobacco lines after incubation with *M. incognita*. Transient transformations of full-length and TN structural domains of *PsoRPM3* have induced significant hypersensitive responses (HR), suggesting that TIR domain might be the one which caused HR. Further, yeast two-hybrid results revealed that the full-length and LRR domain of *PsoRPM3* could interact with the transcription factor *Pso9TF*. The addition of *Pso9TF* increased the ROS levels and induced HR. Thus, our data revealed that the LRR structural domain of *PsoRPM3* may be associated with signal transduction. Moreover, we did not find any relative inductions of defense-related genes *PsoEDS1*, *PsoPAD4* and *PsoSAG101* in *P. sogdiana*, which has been incubated with *M. incognita*. In summary, our work has shown the key functional domain of *PsoRPM3* in the regulation of defense responses to *M. incognita* in *P. sogdiana*.

## 1. Introduction

Root-knot nematodes (*Meloidogyne* spp.) are widespread throughout the world and can parasitize more than 3000 species of plants. Each year, root-knot nematodes cause around 5% of all agricultural losses worldwide [1,2]. However, because root-knot nematodes have a short life cycle and high reproduction rate, they are particularly difficult to control, and the methods used to control root knot nematodes in production need to be further explored. In terms of chemical control, commonly used organic phosphate chemical nematicides, such as sebufos, arbofuran and fenamiphos [3], are non-specific and harmful to humans, birds and fish. In recent years, they have been removed from the pesticide market. The ozone-depleting methyl bromide fumigation method is also being eliminated, and the use of biological fumigation instead of harmful methyl bromide to control nematodes is becoming more and more popular [4]. Another potential method for controlling plant parasitic nematode is microbial preparations, such as bacteria [5], fungi [6] and actinomycetes [7], which have certain control effects on root-knot nematodes, among which *Pseudomonas fluorescens* and *Streptomyces avermitilis* are commercially used for the control of plant parasitic nematodes in many countries [8,9]. The current methods for controlling nematodes are either harmful to the environment or costly, and are not very friendly to agricultural production. With the gradual development of modern molecular biology technology, it is necessary to choose resistant rootstocks or cultivate resistant stocks. Resistant crops are an effective way to control root-knot nematode disease [10].

Most disease-resistance genes in plants have nucleotide-binding-leucine-rich repeat structures (NLRs). Each structural domain plays a different role in plant disease resistance. The N-terminal CC or TIR domain is associated with signaling transduction [11] and is involved in the protein–protein interactions [12,13], which usually self-associate or form complexes through interacting with other proteins [14,15,16]. The intermediate NB-ARC domain is a nucleotide-binding domain that activates or inhibits NLR protein structures by binding to or hydrolyzing ADP/ATP, and is a molecular switch that regulates the activity of NLRs and has a broad role in influencing the self-conjugation of the N-terminal CC or TIR structural domain [17,18,19,20,21]. The C-terminal LRR structural domain is usually responsible for recognizing effectors, preventing auto-activation or auto-inhibition of downstream signals, and is associated with the strength of the immune response [22]. Deletion of the LRR structural domain in some NLRs can lead to autoimmune responses [23,24,25]. The interaction of different structural domains of NLR determines the structure and activity of NLR, resulting in different functions of individual structural domains. However, there are also differences in the functions of the different structural domains for specific NLR proteins, suggesting that the NLR has extremely complex properties. *Mi-1.2* [26], *Mi-9* [27], *CaMi* [28] and *Ma* [29] are resistant to root-knot nematodes, while *Hero-A* [30], *Gpa2* [31] and *Gro1–4* [32] are resistant to cyst nematodes. *Gro1–4* and *Ma* encode TNL, while others encode CNL, in which the Ma protein has a highly polymorphic LRR region, which is considered to be very important for the recognition of PPN (plant parasitic nematode) [33]. In addition, 185 genes with TNL domains have been found in the genome of peach that may be resistant to root-knot nematodes. They all have LRR domains, but there are no more in-depth research reports [34].

Transcription factors are the most common proteins that can interact with the NLR and regulate the NLR-mediated resistant and HR responses [35,36]. A *WRKY* domain was found at the C-terminus of the immune receptor *TNL1* of *Prunus* plants conferring resistance against root-knot nematodes [33]. It has been reported that the transcription factor *WRKY* can regulate the expression of defense-related genes in the immunity triggered by the effector [29], and the presence of the *WRKY* domain in *TNL1* indicates that the NLR protein’s ability to recognize nematode invasion is related to the presence of transcription factors [33,37]. Similarly, in Arabidopsis, TIR-NBS-LRR disease-resistant proteins *DSC1* and *WRKY19* typically regulate the resistance of Arabidopsis to *M. incognita* [38]. Therefore, NLR-associated signalings in plant immunity were conducted via recruiting different transcription factors. However, little is known about the downstream components of NLR immune signaling.

It has been shown that a key downstream component of TIR-NLR immune signaling requires EDS1 (enhanced disease susceptibility 1) [39,40], an adipose-like protein that interacts with *PAD4* (phytoalexin deficient 4) and *SAG101* (senescence-associated gene 101) adipose-like proteins, respectively. *EDS1–SAG101* interaction occurs only in the nucleus, whereas *EDS1–PAD4* interaction is present in the cytoplasm and nucleus [41,42]. *EDS1* also facilitates the interaction between *PAD4* and *SAG101* and forms a larger complex [42]. Co-expression of *EDS1* and *PAD4* leads to autoimmunity [43]. *EDS1* can affect basal immunity and ETI processes as well as HR responses in plants [44]. In contrast, *EDS1*, *SAG101* and *PAD4* are all highly conserved in higher plants [45]. However, the mechanisms of activation and signaling between them remain unknown.

In this study, we screened the transcription factor *Pso9TF*, a basic leucine zipper transcription factor A-like gene, which interacts with the *PsoRPM3* protein, based on the *PsoRPM3* gene with resistance to the root-knot nematode (*M.incognita*), obtained by cloning from a single resistant plant of Xinjiang wild myrobalan plum (*Prunus sogdiana*) and using CO-IP assay—*Pso9TF* was named because this gene is ranked ninth in the CO-IP screening results [46]. Further, we observed the formation of giant cells in the root system of tobacco trans-*PsoRPM3* gene using paraffin sections. The resistance of *PsoRPM3* protein to root-knot nematode (*M. incognita*) was further confirmed by observing the changes of reactive oxygen species (ROS) and HR response in leaves of *N. benthamian* through truncated *PsoRPM3* assay. The interactions between different structural domains of *PsoRPM3* and *Pso9TF* were determined by yeast two-hybrid assay, and it was found that *Pso9TF* could influence the disease resistance of *PsoRPM3* protein. We further measured the expression of TNL-related genes *EDS1*, *PAD4* and *SAG101* in Xinjiang wild myrobalan plum (*Prunus sogdiana*) to *M. incognita*.

## 2. Results

### 2.1. Histological Observations of Tobacco Transgenic for PsoRPM3

**Figure 1:**
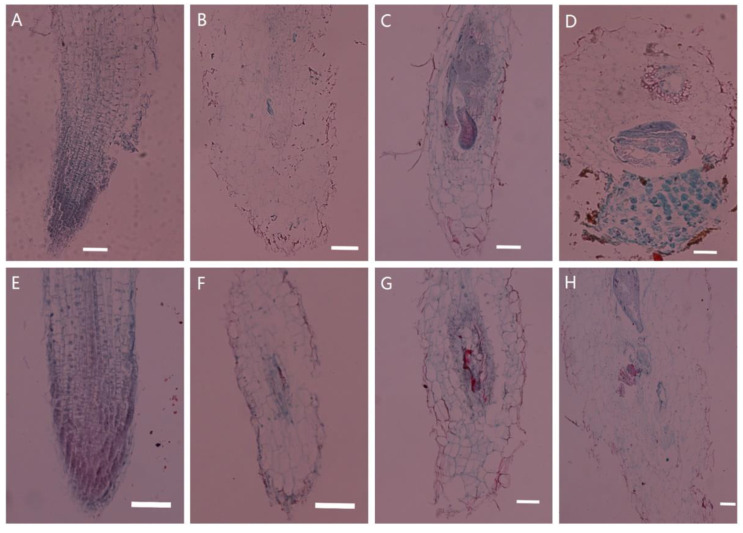
Tissue section to observe the disease resistance response of *PsoRPM3* transgenic tobacco after nematode inoculation. The paraffin section technique was used to observe the nematode invasion status of tobacco roots infected by *M. incognita,* and to compare the difference in disease resistance between the transgenic and wild-type tobacco W38. (**A**–**D**) The observation of wild-type tobacco W38 roots which were inoculated with *M. incognita* at 0 d, 3 d, 14 d and 30 d as a negative control; (**E**–**H**) The observation of transgenic *PsoRPM3* tobacco roots which were inoculated with *M. incognita* at 0 d, 3 d, 14 d and 30 d; scale bar is 100 μm.

Changes in tissue structure were observed after inoculation of wild-type and *PsoRPM3*-transformed tobacco with the *M. incognita*, as shown in Figure 1. The root cells of wild-type and *PsoRPM3*-transformed tobacco developed normally (Figure 1A,E) before inoculation with *M. incognita*. After 3 days post-infection (dpi), a large number of J2 nematodes could be observed in wild-type tobacco roots invading vascular tissues, and there was an expansion of the nematodes (Figure 1B). At 14 dpi, the feeding sites were large, occupying almost the entire longitudinal section of the root system, and each giant cell in the feeding site was cytoplasmically dense, by which time the nematodes had changed to third instar nematodes (Figure 1C). At 30 dpi, the nematodes were already laying eggs and discharging egg masses in large numbers outside the roots (Figure 1D). Invasion of nematodes was also observed in the vascular tissue of a small number of roots at 3 dpi in tobacco transgenic for *PsoRPM3* (Figure 1E). Feeding sites were also formed in some roots at 14 dpi, but the development of giant cells was not full (Figure 1F). Only a few nematodes in the roots became J4 stage at 30 dpi, but no eggs were laid. At this time, the number of giant cells in the roots was low and the development of feeding sites was incomplete (Figure 1G).

### 2.2. Disease Resistance Analysis of the PsoRPM3 Protein

**Figure 2:**
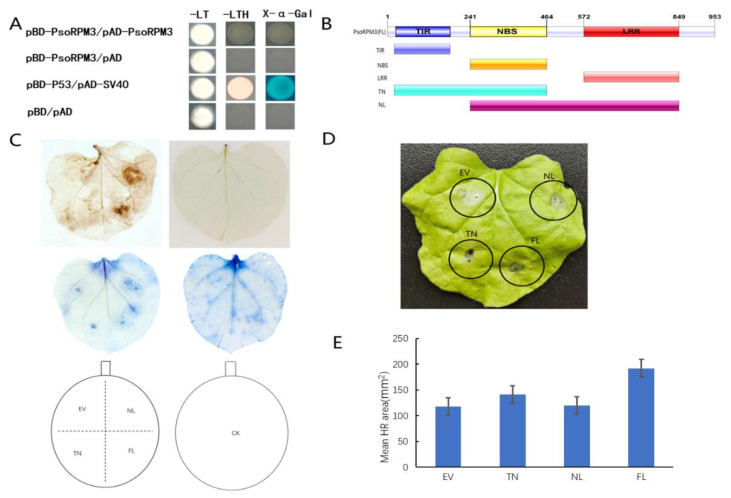
ROS (Reactive oxygen species) burst detection and cell death staining in the *PsoRPM3* disease resistance response. (**A**) Analysis of interactions between *PsoRPM3* and *PsoRPM3* using the yeast two-hybrid system. α-Galactosidase activity was detected using X-α-Gal as a substrate. (**B**) Segmentation pattern of the structural domain of *PsoRPM3* protein. (**C**) The brown color is DAB for reactive oxygen species staining in HR reaction, and the blue color is trypan blue for cell necrosis staining. (**D**) HR responses observed in tobacco (*N. benthamiana)* leaves transiently overexpressing empty vector, TN, NL, and FL 1 d after inoculation with *M. incognita*. (**E**) Determination of cell necrosis area to (**D**).

Although the resistant role of *PsoRPM3* protein to *M. incognita* has been identified, the mode of action of *PsoRPM3* remains unclear. Firstly, we analyzed whether *PsoRPM3* proteins could form homodimers by yeast two-hybrid system, and the results showed that *PsoRPM3* proteins could not form homodimers (Figure 2A). We further divided the *PsoRPM3* protein into full-length, TIR+NBS (TN) and NBS+LRR (NL) domains (Figure 2B) to analyze their respective roles in the plant defense responses, for instance, the ROS (reactive oxygen species) burst and HR. When tobacco leaves were injected with empty vectors, no ROS bursts were detected on the leaves. When the *PsoRPM3* FL (full-length), TN and NL structural domains were injected separately, the full length of *PsoRPM3* protein produced significant ROS production, while both the TN and NL structural domains also produced ROS, but the NL structural domain produced more ROS than the TN structural domain (Figure 2C). Further, we constructed overexpression vectors for transient injection into tobacco leaves to observe the HR response. We found that no HR response occurred in the no-load and a weak HR response occurred in the NL domain, while the TN domain and the full-length *PsoRPM3* protein elicited a significant HR response (Figure 2D). Statistical analysis of the necrotic area of cells eliciting HR responses revealed that the TN structural domain and *PsoRPM3* full-length (FL) mean necrotic area was larger, while the NL structural domain had a slightly larger necrotic area than the empty vector (Figure 2E).

### 2.3. Interaction of PsoRPM3 Protein with Downstream Transcription Factor Pso9TF

**Figure 3:**
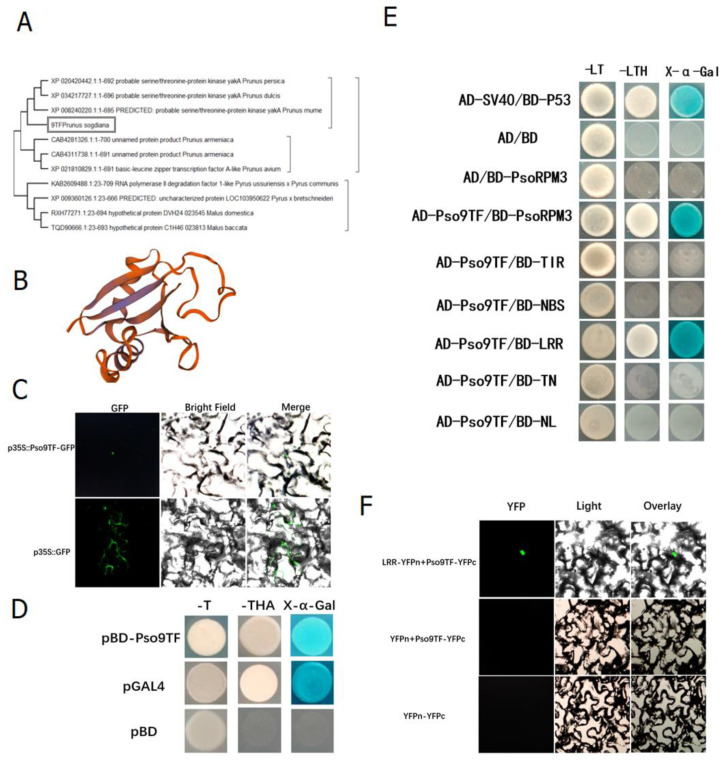
Basic characteristics of *Pso9TF* and detection of interactions with *PsoRPM3*. (**A**) Phylogenetic tree containing *Pso9TF* (rectangle highlighted) and homologous proteins from other related plant species. (**B**) The predicted three-dimensional structures of the *Pso9TF*. (**C**) *Pso9TF-GFP* localized in the nucleus of *N. benthamiana* cells, GFP alone localized throughout the whole cells. Fluorescence (left), bright field (middle), and merged images (right) were obtained at 48 h post-agroinfiltration by using Leica confocal microscopy. (**D**) Transcriptional self-activation of *Pso9TF* using a yeast one-hybrid. (**E**) Interaction of *Pso9TF* with each structural domain of *PsoRPM3* using yeast two-hybrid. (**F**) Interactions between *PsoRPM3* and its three chaperonin proteins were determined by BiFC. YFP fluorescence in the upper epidermis cells of *N. benthamiana* leaves was detected by laser scanning confocal microscopy.

The above studies have shown that *PsoRPM3* protein could activate the production of ROS and induce HR responses, for which the components downstream of the disease resistance signal were not clear. We screened a number of complexes bound to *PsoRPM3* protein in Xinjiang wild myrobalan plum (*P. sogdiana*) by CO-IP based on its localization in the nucleus, including a transcription factor *Pso9TF*. We cloned the *Pso9TF* gene from *P. sogdiana*. The phylogenetic tree analysis revealed that *Pso9TF* was a basic leucine zipper transcription factor or kinase (Figure 3A), and its protein structure contained the PB1 structural domain (Figure 3B). The subcellular localization result showed that *Pso9TF* was localized in the nucleus (Figure 3C). The transcriptional self-activation assay revealed found *Pso9TF* has transcriptional self-activation activity (Figure 3D). Further, yeast two-hybrid assays showed that there was an interaction between *Pso9TF* and the full-length (FL) and LRR structural domains of the *PsoRPM3* protein, but not with the TIR, NBS, TN and NL structural domains (Figure 3E). Their relationships were also verified by BiFC assays (Figure 3F).

### 2.4. Pso9TF and PsoRPM3 Proteins together Cause Disease Resistance Responses

**Figure 4:**
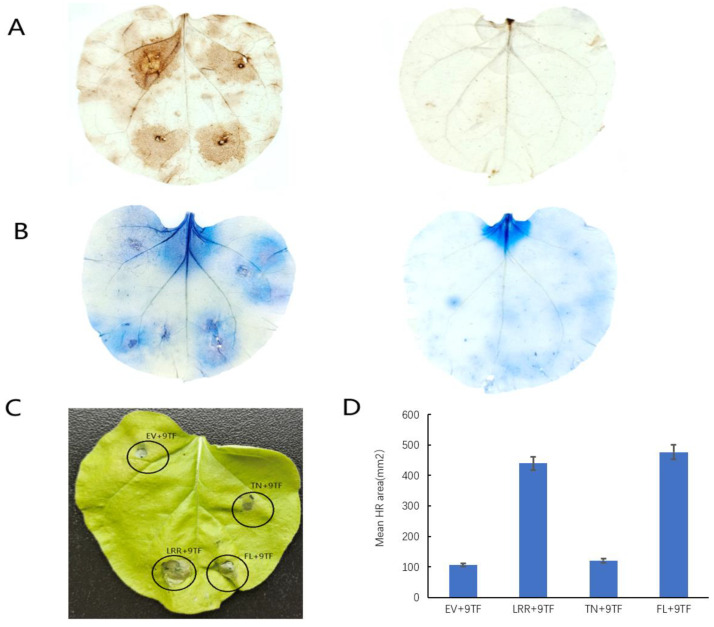
Effect of *Pso9TF* on *PsoRPM3* disease resistance. (**A**) *Pso9TF* and GFP empty vectors, *Pso9TF* and TN, *Pso9TF* and LRR, *Pso9TF* and FL were transiently co-expressed on tobacco leaves of *N. benthamian*, and then injected into nematodes after 1 d, and then stained with DAB for reactive oxygen species. (**B**) Samples stained with trypan blue for necrotic cells; the left was the test group, the right was the control group. (**C**) HR reaction observed directly without any dyeing treatment. (**D**) Measurement and counting of the area of the HR response.

Due to the reciprocal relationship between *PsoRPM3* protein and *Pso9TF*, a question is proposed: do they have a joint effect on disease resistance? The injection of *PsoRPM3* protein full-length followed by *Pso9TF* on tobacco leaves has enhanced the production of ROS and HR response, and the same results were observed for the LRR structural domain of *PsoRPM3* protein, but the TN structural domain did not change much (Figure 4).

### 2.5. Expression of Pso9TF and Related Disease Resistance Genes in Xinjiang Wild Myrobalan Plum

**Figure 5:**
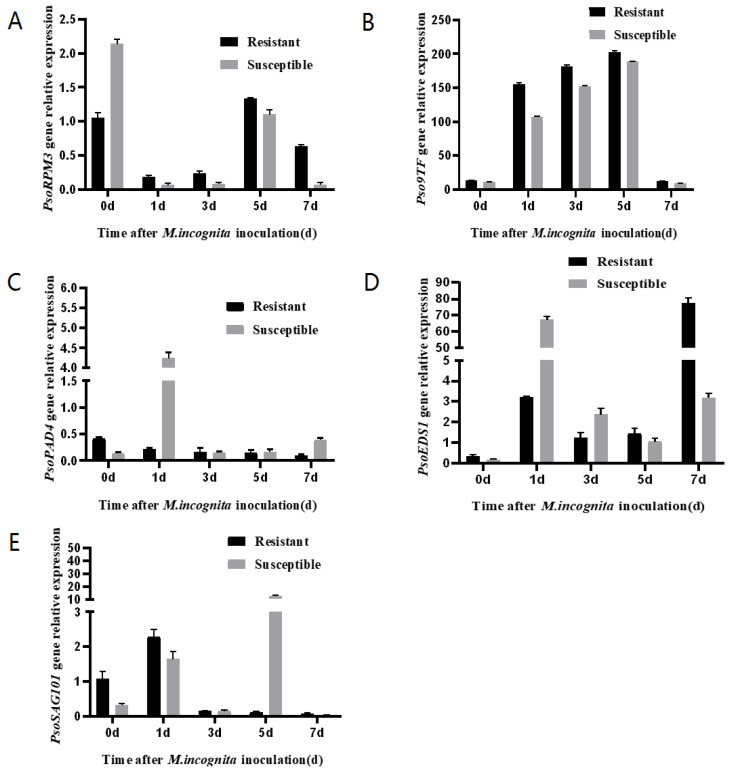
Expression of *Pso9TF* on downstream disease-resistance related genes. (**A**–**E**) Expression of *PsoRPM3*, *Pso9TF*, *PsoPAD4*, *PsoEDS1* and *PsoSAG101* in the roots of resistant and susceptible Xinjiang wild myrobalan plum (*Prunus sogdiana*) individuals at 0, 1, 3, 5, and 7 d post-infection. Real-time PCR data were calculated based on three biological and three technical replicates. Error bars = SD.

Expression of *PsoRPM3*, *Pso9TF*, *PsoPAD4*, *PsoEDS1* and *PsoSAG101* genes was analyzed using roots of Xinjiang wild myrobalan plum cuttings after inoculation with *M. incognita*. The results showed that the expression of the *PsoRPM3* gene increased continuously from 1 to 5 days after inoculation, with the highest on the fifth day (Figure 5A); the *Pso9TF* gene was abundantly expressed in both disease-resistant and susceptible plants from 1 to 5 days after inoculation, but the expression was significantly higher in disease-resistant plants than in susceptible plants, while the expression of *Pso9TF* gene decreased significantly before and 7 days after inoculation (Figure 5B). Both *PsoPAD4* and *PsoEDS1* genes strongly expressed in susceptible plants in different time points (Figure 5C,D). The *PsoSAG101* gene was only expressed in susceptible plants at 5 days after inoculation and was expressed at low levels in the rest of the period (Figure 5E). In all the tested time points, we found that the expression levels of *Pso9TF* and *PsoRPM3* have shown a similar trend: both genes showed significantly more induction in resistant plants than susceptible plants.

## 3. Discussion

The formation of giant cells and the establishment of feeding sites are necessary steps for the successful invasion of root-knot nematodes (*M. incognita*) on plant roots. The giant cells can provide nematodes with various nutrients and support their growth and development by intranuclear replication [47]. The giant cells are surrounded by bast and xylem and cortical cells which gradually produce swollen root knots in the roots [48]. Our observations of the *PsoRPM3*-transformed tobacco roots showed fewer giant cells in the transgenic roots than the control, and giant cell development was delayed, ultimately leading to a failure to form eggs. These findings indicated that the *PsoRPM3*-transformed tobacco significantly improved resistance to *M. incognita*, consistent with previous results of functional validation of the *PsoRPM3* gene [46].

The *PsoRPM3* gene belongs to the TIR-NBS-LRR class of resistance proteins, and there are functional differences between their different structural domains. Studies in *Arabidopsis* have shown that the TIR of *RPS4* and *RRS1* can form homodimers and heterodimers for signaling transduction [49]. The TIR domain of the ZAR1 disease resistance protein forms homodimers and then transduces signals through enzymatic cleavage functions [50]. The TIR domain is thought to function primarily as an articulatory sub-structural domain that mediates protein interactions [51]. The *PsoRPM3* protein did not self-associate in this study, suggesting that *PsoRPM3* may not be involved in the disease resistance process through homodimerization. The full-length, TIR+NBS (TN) and NBS+LRR (NL) structural domains of *PsoRPM3* all induced the production of reactive oxygen species, but only the full-length and TN structural domains of *PsoRPM3* induced a strong HR response, while the NL structural domain caused an inadequate HR response. Our data suggest that the structural domains that induce the HR response are mainly TIR domains.

Resistance proteins generally work together and form complexes in the process of disease resistance in plants. Transcriptional regulators are the most commonly observed interacting proteins with NLRs [52]. Most of these interactions occur in the nucleus, contributing to the accumulation of NLR or transcription factors in the nucleus, regulating the expression of defense genes and thus influencing defense signaling [53,54]. Previous studies have shown that the combination of transcription factors with NLR protein can regulate disease resistance. For example, the combination of rice NLR protein *Pb1* and transcription factor *WRKY45* in the nucleus will stimulate disease resistance [54].Rice *BPH14* is compounded with *WRKY46/WRKY76*, and the combination of the *WRKY46*/*WRKY76* will enhance the expression of the defense gene *RLCK281* and the immune response [55].

The NLR modulates immune signaling by recruiting different transcription factors [53]. The *Pso9TF* transcription factor that interacted with *PsoRPM3* in this study contained the PB1 structural domain, the *PsoRPM3* full-length and LRR structural domains interacted with *Pso9TF*, and both *PsoRPM3* and *Pso9TF* were localized in the nucleus [46], indicating that this interaction occurred in the nucleus. In a transient tobacco injection assay, the full-length (FL) and LRR domains of *PsoRPM3* induced an increase in ROS and an HR response after the addition of *Pso9TF*, suggesting that the LRR domain of *PsoRPM3* may be involved in signal transduction and the HR response.

For most plants, the *EDS1* gene is required downstream of immune signaling by TNLs [39,40,56]. In the Arabidopsis, *EDS1* and *PAD4* genes work closely to stimulate the production of the defense hormone salicylic acid (SA) [57] to limit the growth of pathogens, which is very important for the ETI immune response [58]. Further analysis of Arabidopsis mutants showed that *EDS1* and *PAD4* are also involved in disease resistance controlled by other types of intracellular receptors or pathogen proteins [59]. This indicates that *EDS1* protein usually works with *PAD4* as a regulatory ‘node’ to coordinate the plant’s immune response to various environmental stimuli [57]. It has been reported that the antagonism of the *EDS1/PAD4* complex on the transcription factor *MYC2* enhances the *Arabidopsis* effector to trigger the salicylic acid defense in immunity [60], although *PAD4* can bind to *EDS1* to trigger defense in all aspects of the response, EDS1 can also interact with *SAG101* in the absence of *PAD4* [61]. The *EDS1*–*SAG101* complex is also necessary for TNL-mediated immunity in *N. benthamian* [62]. In addition, studies have shown that chickpea *CaRGA* protein interacts with *WRKY64* in the nucleus to positively regulate EDS1 transcription and cell death signaling [63]; furthermore, in studies of resistance to soybean cyst nematodes, the downstream signaling of *PAD4* is involved in regulating the salicylate signaling pathway and, thus, positively regulates resistance to cyst nematodes [64]. In *P.* *sogdiana*, *PsoEDS1* was abundantly expressed in disease-resistant plants 7 days after inoculation, whereas the expression of *PsoPAD4* and *PsoSAG101* did not change significantly, suggesting that the disease resistance response generated by *PsoRPM3* through interactions with *Pso9TF* did not directly affect the changes in the *PsoEDS1*, *PsoPAD4* and *PsoSAG101* genes. In Arabidopsis, *PAD4* and *SAG101* generally interact with each other to form a complex, thereby affecting the transmission of disease resistance signals. In this study, we found that the resistance of these two genes and *PsoRPM3* to *M. incognita* was not clear. The synergistic relationship indicates that the disease resistance signal pathway of Xinjiang wild myrobalan plum may be different from that of *Arabidopsis.* After the *PsoRPM3* gene initiates disease resistance, the signal pathway needs to be further studied. In the follow-up research, we will further explore other related genes.

Taken together, the current study investigated the structure and function of the screened nematode *M. incognita*-resistant *PsoRPM3* gene and its reciprocal transcription factor *Pso9TF*. Moreover, the molecular mechanism of nematode resistance in Xinjiang wild myrobalan plum (*P.* *sogdiana*) mediated by *PsoRPM3* and *Pso9TF* was clarified in the study, providing an adequate theoretical basis for the selection of nematode-resistant rootstocks. Moreover, this study uses wild rare nematode-resistant rootstocks as test materials and combines transgenic, yeast double hybrid and other molecular biology techniques to further elucidate the molecular mechanism of the nematode-resistant gene *PsoRPM3*, which benefits the selection of disease-resistant rootstocks and the development of the fruit industry.

## 4. Materials and Methods

### 4.1. Plant Materials and Inoculation with M. incognita

Xinjiang wild myrobalan plum (*Prunus sogdiana*) seedlings were planted at the Shangzhuang test station at the China Agricultural University. Resistant and susceptible Xinjiang wild myrobalan plum individuals were identified after inoculation with root-knot nematode (*M. incognita*) over eight consecutive years (2007–2015). Robust shoots were harvested yearly in early May and sliced into 15–20 cm cuttings, with an upper horizontal cut and a lower diagonal cut, with each cutting bearing only 1–2 leaves. The cuttings were rooted in a 1:1 mixture of perlite and vermiculite.

Root-knot nematodes (*M. incognita*) were sourced from the laboratory of Jian Heng from the Institute of Plant Protection of the China Agricultural University. Nematode cultures were maintained according to a previously published method [65] with slight modifications: eggs were collected from susceptible tobacco W38 roots, placed on nylon netting floating in water, and maintained in darkness at 30 °C for 5 days, at which point juvenile nematodes (J2) were collected for analysis.

Xinjiang wild myrobalan plum seedlings were inoculated with root-knot nematode (*M. incognita*). Seedlings at similar stages of growth were inoculated with nematodes, using 2000 J2 *M. incognita* per seedling. Five resistant and five susceptible seedlings were irrigated with identical amounts of water to serve as experimental controls. All of the root tips were collected at 0, 1, 3, 5 and 7 days post-infection (dpi) for gene cloning and expression assays.

Seedlings of tobacco W38 (*Nicotiana tabacum cv.* W38) and *Nicotiana benthamiana* were cultured in a light incubator at 24–25 °C. The *PsoRPM3*-transformed tobacco seedlings were grown in sterile glass flasks to the height of about 25 cm and then transplanted into soil substrate in a light incubator at 24–25 °C.

### 4.2. Gene Cloning and RT-qPCR Assays

Total RNA was isolated from Xinjiang wild Myrobalan plum roots using an EASY Spin Kit (Beijing Biomed Biotechnology Co., Ltd., Beijing, China) and used to prepare cDNA using oligo-dT18 primers (Takara Biomedical Technology Co., Ltd., Beijing, China). Using the data of protein XP_021810829.1, which was screened and compared in the gene bank of plum genus using CO-IP results, primers were designed in the 5′UTR and 3′UTR regions to clone *Pso9TF* using cDNAs from the root of disease-resistant Xinjiang wild myrobalan plum as templates. We performed quantitative real-time PCR (qRT-PCR) with the cDNAs on an ABIPRISM 7500 (Thermo Fisher Scientific, Waltham, MA, USA) based on the manufacturer’s instructions, with 40 cycles of 95 °C for 10 s and 60 °C for 30 s, using SuperReal PreMix Plus (Tiangen Biotech Co., Ltd., Beijing, China). The relative RNA abundance was calculated using the 2^△△CT^ method [66], using *PsoRPII* as the reference gene [67]. All primers were designed using Primer 5.0 software. The primers of *PsoRPM3*, *Pso9TF*, *PsoEDS1*, *PsoPAD4*, and *PsoSAG101* in this study were listed in Supplementary Table S1.

### 4.3. Paraffin Sectioning and Histological Observation

The roots of wild tobacco W38 and transgenic *PsoRPM3* tobacco were collected after inoculation with 2000 J2 *M. incognita* for 0 d, 3 d, 14 d, 30 d; then, the roots were removed from the soil and cleaned with water. The samples were fixed in paraformaldehyde (FAA), dehydrated, embedded, sectioned, and stained with Safranin Fast Green dye prior to microscopy. Refer to reference [68] for the specific method of the test.

### 4.4. Protein Location Analysis of Pso9TF

Using the 35S promoter-driven *Pso9TF-GFP*, Agrobacterium-mediated transient expression was performed in *N. benthamian* leaves, with 35S: GFP serving as a negative control. The Agrobacterium strain transformed with the vector was cultured in YEP medium, harvested, and diluted to OD_600_ = 0.6–0.8, and then infiltrated into *N. benthamian* leaves using a syringe without a needle. At 48 h post-injection, infected leaves’ epidermis were analyzed under an Olympus BX61 fluorescence microscope (Olympus Corporation, Tokyo, Japan).

### 4.5. The Hypersensitive Response in Tobacco Leaves

A 35S-driven *PsoRPM3* overexpression vector was constructed for Agrobacterium-mediated transient expression. Tobacco leaves were infiltrated with the Agrobacterium of *p35S: GFP* or full-length *p35S: PsoRPM3-GFP* or several structural domains of *p35S: PsoRPM3-GFP* or with *Pso9TF* after 24 h inoculation. Approximately 300 nematodes were injected into each treatment on the leaves of tobacco (*N. benthamian*), and their respective necrotic areas were analyzed in ImageJ software (https://imagej.nih.gov/ij/ accessed on 9 January 2021). The number of replicates per treatment was at least 30 leaves.

Reactive oxygen species (ROS) staining referred to the method in this literature [69]; the necrotic cells were stained with trypan blue, and the test method referred to in [70].

### 4.6. Yeast Two-Hybrid Analysis

Yeast two-hybrid analysis was conducted using the yeast strain AH109. The bait vector used was pGBKT7, and the prey vector was pGADT7. Competent yeast cells were transformed with specific vector combinations using the freeze–thaw method. The AH109 yeast strain was grown on SD/-Leu-Trp medium for 2–3 days at 30 °C, and 9 independent clones were picked and cultured on SD/-His-Leu-Trp medium at 30 °C for 2–3 days. Then, they were analyzed for α-galactosidase activity via color development using X-α-Gal (4 mg/mL, 5 µL per colony).

### 4.7. Bimolecular Fluorescence Complementation (BiFC)

The coding sequences of *PsoRPM3*-LRR and *Pso9TF* were amplified and individually inserted into the vector pUC-SPYNE-YFPn and pUC-SPYNE-YFPc. Each vector was transformed into *Agrobacterium tumefaciens* GV3101 and cultured for 2–4 days on YEP plates containing kanamycin and rifampin. Each positively transformed colony was cultured in YEP liquid medium overnight, harvested by centrifugation, and resuspended in inducing buffer (10 mM MgCl_2_, 0.2 mM acetosyringone, and 10 mM MES, pH 5.6) to a final concentration of OD_600_ = 1.0. After four hours, bacteria harboring YFPn-*PsoRPM3*-LRR and YFPc-*Pso9TF* were mixed at a ratio of 1:1 and infiltrated into the *N. benthamiana* leaves for transient expression. At 48 h post-infection (hpi), the fluorescence of the *N. benthamiana* epidermal cells was imaged using a confocal laser fluorescence microscope (Olympus BX61).

## Supplementary Materials

The following are available online at https://www.mdpi.com/article/10.3390/plants10081561/s1, Table S1: Primers used in this study.
