# Supplementary material for: Transcription Factor Pso9TF Assists Xinjiang Wild Myrobalan Plum (Prunus sogdiana) PsoRPM3 Disease Resistance Protein to Resist Meloidogyne incognita
Source: Plants (Basel). 2021 Jul 29;10(8):1561. doi: 10.3390/plants10081561 (PMC8402125; doi:10.3390/plants10081561)
Supplement: Supplementary file 1 [file plants-10-01561-s001.zip › plants-1271506-supplementary.pdf]

**Supplementary Table S1** Primers used in this study

| Name | Sequence (5‘→3‘) | Purpose |
|---|---|---|
| *PsoRPII-F* | TGAAGCATACACCTATGATGATGAAG | RealTime-PCR Specific |
| *PsoRPII -R* | CTTTGACAGCACCAGTAGATTCC | houskeeping |
| *Pso9TF -F* | TATTGGGATAGAGGAACAGT | RealTime-PCR Specific |
| *Pso9TF -R* | GCAAAGGTAATGGCAGAG | *Pso9TF* |
| *PsoRPM3 -F* | AGACCAAGTTGGAGTGGATAGCA | RealTime-PCR Specific |
| *PsoRPM3-R* | CCGTCAAGGCTTATTCTGAGTAT | *PsoRPM3* |
| *PsoEDS1 -F* | GAAGAAGCAGGAGCAGTCGT | RealTime-PCR Specific |
| *PsoEDS1-R* | CCACAGAAGCTTGAAATGAGGT | *PsoEDS1* |
| *PsoPAD4-F* | TATGGTCGACGCTGCCATAC | RealTime-PCR Specific |
| *PsoPAD4-R* | CACGTGGCAGAAGTTGTGTG | *PsoPAD4* |
| *PsoSAG101-F* | CTGCTCCGAGAACAACCCAT | RealTime-PCR Specific |
| *PsoSAG101-R* | ACCCGAATCATGAACACCGT | *PsoSAG10* |
| *Pso9TF-full-F* | ATGGATCCACCACCTCTCCCACCTC | Full length *Pso9TF* gene |
| *Pso9TF-full-R* | TTATGTCGGTAGCTGCTGTTTCG | cloning primer |
